# The Association between Neighborhood Amenities and Cognitive Function: Role of Lifestyle Activities

**DOI:** 10.3390/jcm9072109

**Published:** 2020-07-04

**Authors:** Osamu Katayama, Sangyoon Lee, Keitaro Makino, Ippei Chiba, Seongryu Bae, Yohei Shinkai, Kenji Harada, Hiroyuki Shimada

**Affiliations:** Department of Preventive Gerontology, Center for Gerontology and Social Science, National Center for Geriatrics and Gerontology, 7-430 Morioka-cho, Obu City, Aichi 474-8511, Japan; sylee@ncgg.go.jp (S.L.); kmakino@ncgg.go.jp (K.M.); ichiba@ncgg.go.jp (I.C.); bae-sr@ncgg.go.jp (S.B.); yshinkai@ncgg.go.jp (Y.S.); harada-k@ncgg.go.jp (K.H.); shimada@ncgg.go.jp (H.S.)

**Keywords:** lifestyle activity, neighborhood amenities, mild cognitive impairment, global cognitive impairment, older adults

## Abstract

Many of the modifiable risk factors for dementia are lifestyle-related, and multidomain interventions tailored to individual lifestyles are recommended to prevent cognitive decline and dementia. However, studies of the relationship between the environment and cognitive function have shown that cognitive disorders and dementia are more prevalent in rural areas than in urban areas. The purpose of this study was to clarify the role of lifestyle activities on the association between neighborhood amenities and cognitive function. Our data were measured between August 2011 and February 2012. Participants comprised 3786 older adults (mean age: 71.5 years, standard deviation (SD) = ±5.2). We categorized neighborhood amenities as institutional resources that promote cognitively beneficial activities such as physical activity. We calculated the Walk Score^®^ for all participants using their home address and divided them into three groups. We assessed their 12 lifestyle activities performed outdoors. Cognitive function was measured via Mini-Mental Status Exam, word list memory, attention, executive function, and processing speed. We found that participants who were more likely to report many lifestyle activities were more likely to have normal cognition, even in areas where neighborhood amenities were scarce. The clinical significance of this study is that increased lifestyle activity contributes to the prevention of cognitive decline.

## 1. Introduction

The increase in aging populations worldwide has been accompanied by a rise in the prevalence of dementia and Alzheimer’s disease (AD) [1], causing health, economic, and social burdens [2,3]. A 2014 study reported that there would be 46.8 million people with dementia worldwide by 2015, and that the number was projected to double every 20 years, reaching 74.7 million in 2030 and 131.5 million in 2050 [4]. Thus, reducing the incidence of dementia is a global necessity. Interventions involving modifiable risk factors for dementia in middle-aged (45–65 years) and older adults (65 years and older) without dementia may delay or prevent dementia in one third of people [5]. Multidomain interventions tailored to individual lifestyles have been recommended to prevent cognitive decline and dementia because many of the modifiable risk factors for dementia are lifestyle-related [6]. Practice of multidomain lifestyle activities has been shown to be associated with an improvement from mild cognitive impairment (MCI) to normal cognitive function [7]. A person with a more active lifestyle is less likely to develop cognitive decline and dementia than a less active person [8,9,10,11].

Meanwhile, previous studies of the relationship between environment and cognitive function have shown that cognitive disorders and dementia are more prevalent in rural areas than in urban areas [12]. Clarke et al. reported that increasing the density of institutional resources (schools, libraries, communication centers, etc.) that promote cognitively beneficial activities, including physical activity, affects cognitive function, and that urban environments with increased density, may promote cognitive reserve in older people [13]. Additionally, meta-analyses have reported that an early rural life is associated with AD [14]. However, to reduce the incidents of dementia, it is not efficient to relocate older adults who live in a neighborhood with limited resources to an environment with numerous ones. There are reports suggesting that people living in rural areas experience a broader social network than urban dwellers [15,16], and have higher social involvement [17]. Social support may be more important in rural settings, where access to adequate services and facilities is often limited, and can cause people to become dependent upon family and friends for support [16]. Furthermore, individuals with exciting jobs, and more favorable socioeconomic backgrounds have been shown to have a lower risk of dementia in old age [18,19]. Additionally, socioeconomic status has been identified as an important mediator in the relationship between environment and cognitive health in old age [20,21]. Thus, previous studies have identified associations between individuals’ lifestyles, environments, socioeconomic status, and cognitive outcomes. The results of these studies suggest that an active lifestyle may be the key to achieving a positive effect on cognitive function, even for older adults in areas where neighborhood amenities are rare.

This study refers to “neighborhood affluence” as defined by Clarke et al. [13]. Neighborhood affluence involves institutional resources (e.g., schools, libraries, and community centers) that promote cognitively beneficial activities, including physical activity. Therefore, in this study, “neighborhood affluence” was defined as “neighborhood amenities”. This research utilized Walk Score™ (Front Seat Management, Limited Liability Company (LLC), Seattle, WA, USA) [22], a publicly available website found to be valid and reliable for estimating access to amenities within a comfortable walking distance [23]. Walk Score uses data provided by the Google™ AJAX Search application program interface [24], along with a geography-based algorithm to identify nearby amenities and calculate a “walkability” score [22] based on distance to amenities. Neighborhood walkability has been shown to be associated with physical activity and social participation in older adults [25,26,27]. Therefore, the walk score was utilized as a surrogate marker for neighborhood amenities.

The purpose of this study was to clarify the role of lifestyle activities on the association between neighborhood amenities and cognitive function. It was hypothesized that an active lifestyle could maintain cognitive function, even in areas with limited neighborhood amenities near homes.

## 2. Methods

### 2.1. Study Participants, Design, and Setting

**Figure 1:**
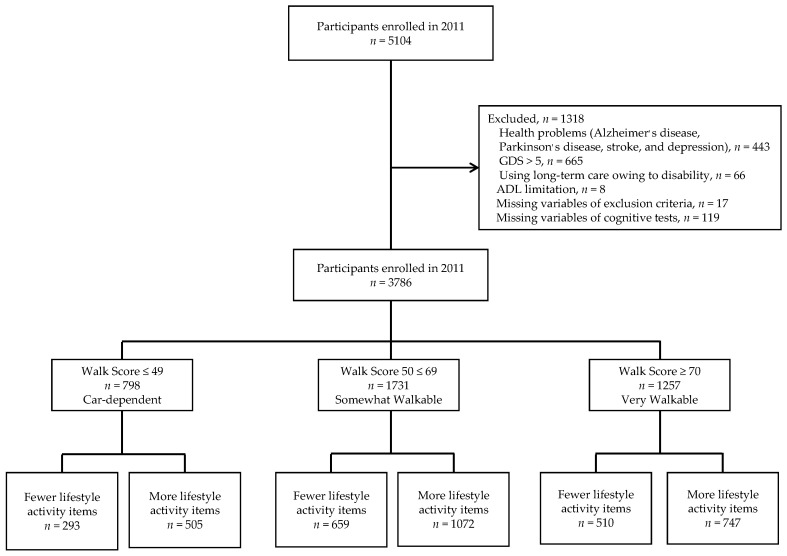
Flow diagram of sample selection. GDS: 15-item Geriatric Depression Scale.

Participants were selected from adults enrolled in a population-based cohort study titled “The Obu Study of Health Promotion for the Elderly” [28], which is part of the National Center for Geriatrics and Gerontology-Study of Geriatric Syndromes (NCGG–SGS), a cohort study with the primary goal to establish a screening system for geriatric syndromes and to validate evidence-based interventions for preventing geriatric syndromes [29]. The Obu Study of Health Promotion for the Elderly enrolled community-dwelling older adults aged 65 years and older. Participants were recruited from Obu, a residential suburb of Nagoya, Japan. Between August 2011 and February 2012, 5104 community-dwelling older adults participated in an assessment that included a face-to face interview, and measures of physical and cognitive function [28]. Exclusion criteria included possessing health problems, such as AD, Parkinson’s disease, history of stroke, or depression (*n* = 443) or depressive symptoms (score of >5 on the 15-item Geriatric Depression Scale) [30] (*n* = 665), need for support or care as certified by the Japanese public long-term care insurance system because of disability (*n* = 66), inability to perform basic tasks of daily living, such as eating, grooming, bathing, and climbing up and down stairs (*n* = 8), missing data regarding the exclusion criteria (*n* = 17), and missing data regarding the cognitive tests (*n* = 119). A total of 1318 potential participants were excluded, resulting in a final sample of 3786 participants, who were included in the 2020 analysis. All participants voluntarily provided informed consent before inclusion. The Ethics Committee of the National Center for Geriatrics and Gerontology approved the study protocol (No. 523, June 30 in 2011) (Figure 1).

### 2.2. Measurement of Neighborhood Amenities

The Walk Score was based on distance to 13 categories of amenities (grocery stores, coffee shops, restaurants, bars, movie theaters, schools, parks, libraries, book stores, fitness centers, drug stores, hardware stores, clothing/music stores) [22]. Each category was weighted equally and points were summed and normalized to yield a score of 0–100 (0: Almost all errands require a car, 100: Daily errands do not require a car) based on the distance from a specific address to the amenities [22]. Walk Score has been shown to be related to locally derived and built environment indices [23,31,32]. The Walk Score was calculated for all participants, using their home address, and they were divided into 3 groups based on the distance to amenities: “car-dependent” (score: 0–49), “somewhat walkable” (50–69), and “very walkable” (70–100).

### 2.3. Measurements of Lifestyle Activity

Questionnaire items were adapted from the Japan Science and Technology Agency (JST-IC) [33], the Kihon Checklist (KCL) [34], and the NCGG–SGS [7,29,35,36]. Data about engagement in 12 lifestyle activities performed outdoors were collected through a questionnaire with the following questions: (1) “Do you go outdoors using the bus and train?” (2) “Do you go to buy daily necessities?” (3) “Do you engage in cash handling and banking?” (4) “Do you go to a friend’s house?” (5) “Do you drive a car?” (6) “Do you go out to discard the trash?” (7) “Do you engage in fieldwork or gardening?” (8) “Do you engage in hobbies or sports activities?” (9) “Do you participate in events at the community center?” (10) “Do you attend meetings in the community?” (11) “Do you use maps to go to unfamiliar places?” and (12) “Do you engage in cultural classes?” The response “yes” was considered a positive answer. The 3 neighborhood amenities groups were then divided into 2 groups each, “Fewer lifestyle activity items” and “More lifestyle activity items,” resulting in a total of 6 groups (See Figure 1). In this study, we divided them by median.

### 2.4. Measurement of Cognitive Functions

To conduct cognitive screening, the National Center for Geriatrics and Gerontology-Functional Assessment Tool, which is an iPad application, was employed [37]. The tool comprises 4 domains: (1) memory (word list memory-I (immediate recognition) and word list memory-II (delayed recall)), (2) attention (a tablet version of the Trail Making Test-part A); (3) executive function (a tablet version of the Trail Making Test-part B), and (4) processing speed (a tablet version of the Symbol Digit Substitution Test). The tool has high test–retest reliability and moderate to high criterion-related validity [37] and predictive validity [38] among community-dwelling older adults. Cognitive assessments were conducted by staff who were trained by the authors. Potential participants were identified with MCI after reviewing the available clinical, neuropsychological, and laboratory data at meetings involving study neurologists and neuropsychologists, as described in a previous study [28]. Briefly, MCI participants were independently assessed using the National Center for Geriatrics and Gerontology-Functional Assessment Tool, which has two memory tasks, attention and executive function tests, and a processing speed task. Using established criteria [39], MCI was diagnosed in individuals who exhibited cognitive impairment but were functionally independent in terms of basic daily life activities. For all cognitive tests, the established standardized thresholds were used in each corresponding domain to define impairment in population-based cohorts comprising community-dwelling older adults (scores of >1.5 standard deviations that specified the age and educational means). Global cognitive function was measured using the Mini-Mental State Examination (MMSE) [40]. Specifically, the MMSE score of <24 points was determined as a cutoff for global cognitive impairment (GCI), conforming to previous findings [41]. Participants whose cognitive test scores were all >1.5 standard deviation units above the mean were categorized within the normal cognition group.

### 2.5. Potential Confounding Factors

Demographic variables and chronic disease are associated with cognitive function in older adults. The multinomial logistic regression model included the following covariates: participant’s age, sex, years of education, and number of medications. Years of education was coded as a continuous variable. Years of education and the number of medications taken were entered into the regression model as continuous variables. The presence of the following self-reported chronic diseases was also entered into the model: hypertension, heart disease, and diabetes. In addition, the median value of the number of items in the 12 lifestyle activities implemented by all participants was calculated.

### 2.6. Statistical Analysis

The normality of data was verified using the Shapiro–Wilk test. The non-parametric Kruskal–Wallis test was used, due to all non-normally distributed scores. Following the Kruskal–Wallis test, the Bonferroni correction for the Mann–Whitney U-test and Pearson’s Χ^2^ test was used to detect the differing characteristics between the neighborhood amenities groups. Residuals followed the *t* distribution wherein *t* > 1.96 indicated *p* < 0.05. Multinomial logistic regression analysis was used to examine the association of cognitive status with neighborhood amenities, and cognitive status was set as the dependent variables (with the most typical, NC as the reference group) after adjusting for covariates. Similarly, following the Kruskal–Wallis test, the Bonferroni correction for the Mann–Whitney U-test, and Pearson’s Χ^2^ test were used to compare the variables between the 6 groups combining neighborhood amenities statuses and lifestyle activity items. Finally, Multinomial logistic regression analysis was used to examine the association of cognitive status with lifestyle activities in each neighborhood amenities group, and cognitive status was set as the dependent variable (with the most typical, NC as the reference group) after adjusting for covariates. Adjusted odds ratios (ORs) and 95% confidence intervals (CIs) were calculated. The significance level was set at *p* < 0.05. All analyses were performed using IBM SPSS version 25.0 (IBM, Tokyo, Japan).

## 3. Results

### 3.1. Characteristics of the Participants

The car-dependent, somewhat walkable, and very walkable groups accounted for 798 (21.1%), 1731 (45.7%), and 1257 (33.2%) participants of the total study population, respectively. Descriptive statistics for all variables, based on neighborhood amenities status are shown in Table 1. Significant differences were observed among the 3 groups with respect to years of education, MMSE score, and number of lifestyle activities (*p* < 0.05).

### 3.2. Association of Cognitive Status with Neighborhood Amenities Status

Table 2 shows the ORs and 95% CIs estimated by unadjusted and adjusted multinomial logistic regression analyses, with cognitive status as the dependent variable and neighborhood amenities groups as the independent variable. After adjusting for potential confounding factors (age, sex, years of education, number of medications, hypertension, heart disease, diabetes, and number of lifestyle activities), car-dependent and somewhat-walkable groups were not independently associated with MCI (OR: 0.99; 95% CI: 0.78, 1.26 and OR: 0.99; 95% CI: 0.82, 1.20 respectively) and GCI (OR: 1.28; 95% CI: 0.97, 1.69 and OR: 1.10; 95% CI: 0.86, 1.39 respectively). Supplementary Table S1 shows the characteristics of the subgroups of cognitive states (NC, MCI, and GCI).

### 3.3. Characteristics of the 6 Groups Combining Neighborhood Amenities Status and Lifestyle Activity Items

Each neighborhood amenities group was divided into 2 groups, one with less than 9 and one with more than 9 lifestyle activity items. The group with fewer lifestyle activity items and the group with more lifestyle activity items accounted for 293 (7.7%) and 505 (13.3%) participants respectively, in the car-dependent group, 659 (17.4%) and 1072 (28.3%) respectively, in the somewhat walkable group, and 510 (13.5%) and 747 (19.7%) respectively, in the very walkable group. Descriptive statistics for all variables of cognitive function in participants grouped combining neighborhood amenities status and lifestyle activity items are shown in Table 3. Significant differences were observed among the 6 groups with respect to years of education, number of medications, MMSE score, Trail Making Test score-parts A and B, and Symbol Digit Substitution Test score (*p* < 0.01).

### 3.4. Association of Cognitive Status with Neighborhood Amenities and Lifestyle Activity Status

**Figure 2:**
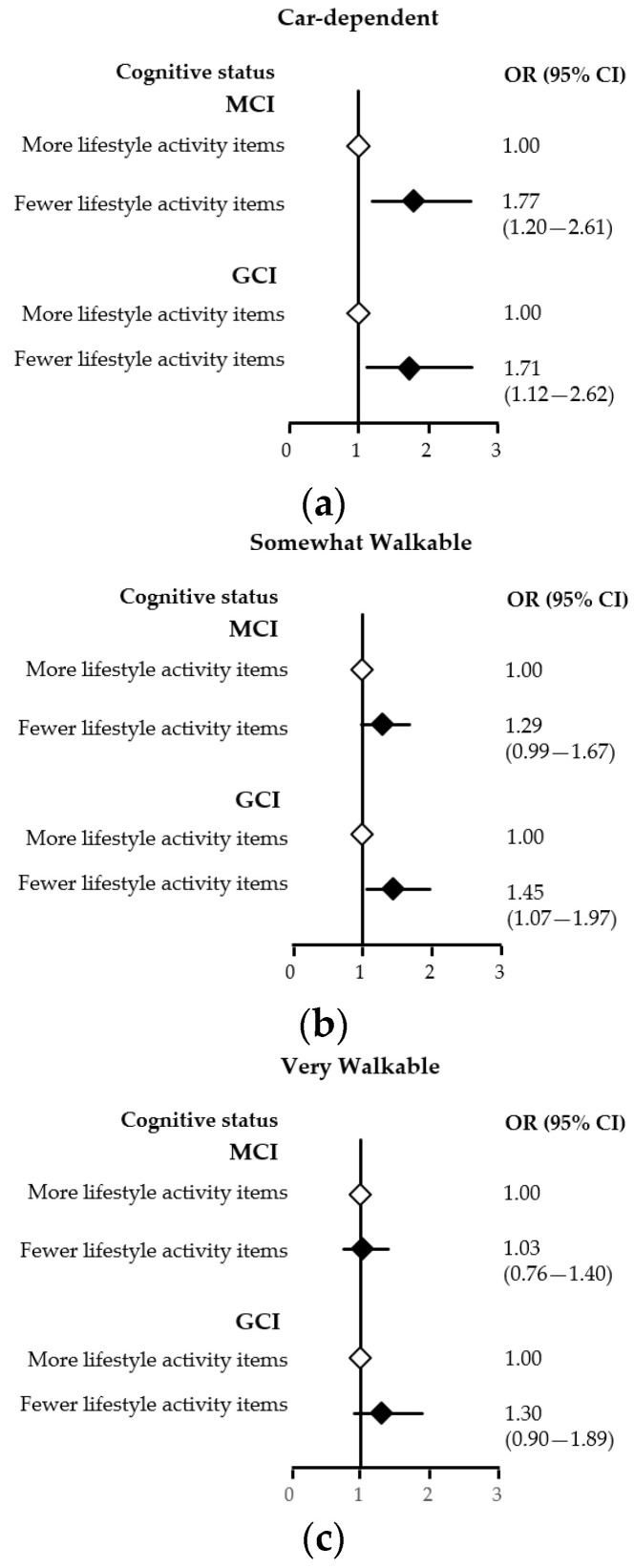
Forest plot showing adjusted associations between neighborhood amenities status and lifestyle activity items and cognitive status in a sample of older adults. CI: confidence interval; GCI: global cognitive impairment; MCI: mild cognitive impairment; OR: odds ratio. The car-dependent group (**a**), the somewhat walkable group (**b**), and the very walkable group (**c**) are shown. The group with fewer lifestyle activity items (hollow diamonds) and the group with more lifestyle activity items (solid diamonds).

Figure 2 shows the ORs and 95% CIs estimated by adjusted multinomial logistic regression analyses in each neighborhood amenities group, with cognitive status as the dependent variable and lifestyle activity status as the independent variable. After adjusting for potential confounding factors in the car-dependent group, the group with fewer lifestyle activity items was independently associated with MCI (OR: 1.77; 95% CI: 1.20, 2.61) and GCI (OR: 1.71; 95% CI: 1.12, 2.62) (Figure 2a). In the somewhat walkable group, the group with fewer lifestyle activity items was not independently associated with MCI (OR: 1.29; 95% CI: 0.99, 1.67), but independently associated with GCI (OR: 1.45; 95% CI: 1.07, 1.97) (Figure 2b). Finally, in the very walkable group, the group with fewer lifestyle activity items was not independently associated with MCI (OR: 1.03; 95% CI: 0.76, 1.40) and GCI (OR: 1.30; 95% CI: 0.90, 1.89) (Figure 2c).

## 4. Discussion

The aim of this study was to clarify the role of lifestyle activities on the association between neighborhood amenities and cognitive function. As initially hypothesized, it was revealed that participants who were more likely to report many lifestyle activities were also more likely to have normal cognition, even in areas where neighborhood amenities were scarce.

The car-dependent, somewhat walkable, and very walkable groups accounted for 798 (21.1%), 1731 (45.7%), and 1257 (33.2%) participants of the total study population. A comparison of the three groups showed that years of education was lowest in the car-dependent group and the MMSE score was significantly higher in the very walkable group compared to the car-dependent group. Existing research has speculated that living in highly educated and socioeconomically advantaged neighborhoods attributes the greater density of physical health resources (recreational centers, gyms, parks, walking paths, health food stores, etc.) with promoting cognitive function and/or buffering cognitive decline [13]. To clarify the relationship between neighborhood amenities and cognitive function (i.e., normal cognition, MCI, and GCI), a multinomial logistic regression analysis was performed with cognitive function as the dependent variable. However, no statistically significant association was found. In recent years, in addition to environmental factors, genetic factors and lifestyle characteristics have been reported as factors related to cognitive function [7,42]. Present results support prior studies that reveal cognitive function is not associated solely with neighborhood amenities, but with multiple factors.

Meanwhile, in the six groups categorized combining neighborhood amenities statuses and lifestyle activity items, the group with more lifestyle activities had longer years of education and performed better than the group with fewer lifestyle activities on the MMSE, Trail Making Test A and B, and the Symbolic Digit Substitution Test. A multinomial logistic regression analysis with cognitive function as the dependent variable revealed that the car-dependent group was significantly associated with MCI and GCI when there were fewer lifestyle activity items. The somewhat walkable group was associated with only GCI in the group with fewer lifestyle activity items. There was no significant association with either MCI or GCI in the very walkable group. Protective factors for dementia include high education and physical activities [9]. The car-dependent group had shorter years of education compared to the somewhat walkable and very walkable groups. Therefore, for the group with fewer lifestyle activities in the car-dependent group, lack of neighborhood amenities and low levels of physical activity and years of education were considered to be factors that were associated with MCI and GCI. The somewhat walkable group had higher neighborhood amenities and longer years of education than the car-dependent group. Therefore, the group with fewer lifestyle activities in the somewhat walkable group was considered to be related to GCI only, with low lifestyle activity, unlike the car-dependent group. Finally, the group with fewer lifestyle activities in the very walkable group was not associated with MCI or GCI. Neighborhood amenities may be particularly important for older adults, who spend more time in the neighborhood in which they live, and are more sensitive to neighborhood characteristics, such as safety and physical access [43,44]. Previous studies have reported that socioeconomic status has been identified as an important mediator in the relationship between environment and cognitive health in old age [20,21]. Socioeconomic status is often associated with cognitive function [45,46]. Furthermore, it has been shown that individuals with higher levels of education, stimulating jobs and advantaged socioeconomic backgrounds are at lower risk for dementia in older age [18,19,47]. Although it is not clear in this study, because we did not interview participants about their jobs or socioeconomic backgrounds, it is possible that walkability, the neighborhood, the home, and longer years of education may have a protective effect on cognitive function in the “very walkable” group.

The strengths of this study include the large sample size and an operationalized assessment to identify neighborhood amenities, lifestyle activity, and cognitive function. However, some limitations are evident. First, the cross-sectional design requires that the causal relationships between neighborhood amenities, lifestyle activity, and cognitive function be clarified in future prospective studies. Second, this study did not use random sampling for data collection; hence, under-reporting of the incidence rate of MCI and GCI among older adults is possible. The participants in this sample were all individuals who were capable of accessing health checkups from their homes, meaning individuals with various other conditions were excluded. In addition, a control group of community-dwelling older adults aged 65 years and older with cognitive impairment was not established, so the relationship between lifestyle activities and cognitive functioning has not been completely clarified. Next, there were several blind spots in the lifestyle activity questionnaire. It was limited in terms of questions regarding transportation when going out, participation in events, and social activities. Further clarification of relevant life activities and transportation mediums in future research could allow clinicians to provide more older adult-centered care, which could result in better outcomes in the primary prevention of cognitive decline. Finally, this study failed to address other covariates related to genetic factors (e.g., ApoEε4), and covariates such as marital status (single, married, divorced or widowed), and health variables (smoking, alcohol use) could also affect cumulative age-related changes. The interview questions, “Are you living alone,” and “How many housemates do you have,” did not inquire as to the relationship with housemates. Therefore, it was impossible to consider the impact of marital status. Naturally, future studies should include these clarifying inquiries. Despite these limitations, the findings are significant in that it was discovered that participants who engaged in numerous lifestyle activities were able to maintain cognitive function, even in residential areas where neighborhood amenities were uncommon. The clinical significance of this study asserts that in areas with fewer neighborhood amenities, the recommendation of increased lifestyle activity would contribute to the prevention of cognitive decline.

## Figures and Tables

**Table 1:** Demographic characteristics of older adults from each neighborhood amenities group.

	Total *n* = 3786	Car-Dependent *n* = 798 (21.1)	Somewhat Walkable *n* = 1731 (45.7)	Very Walkable *n* = 1257 (33.2)	*p*-Value	Post Hoc
Demographic characteristics						
Age, year *	70.0 (68.0–75.0)	70.0 (67.0–74.0)	70.0 (68.0–75.0)	70.0 (67.0–74.0)	0.07 ^a^	
Sex, Female (%)	1948 (51.5)	402 (50.4)	870 (50.3)	676 (53.8)	0.13 ^b^	
Education, years *	12.0 (9.0–12.0)	12.0 (9.0–12.0)	12.0 (9.0–13.0)	12.0 (9.0–12.0)	<0.001 ^a^	Car < Somewhat, Very
Medication, number *	1.0 (0.0–3.0)	1.0 (0.0–3.0)	1.0 (0.0–3.0)	1.0 (0.0–3.0)	0.54 ^a^	
Chronic disease						
Hypertension, no (%)	1681 (44.4)	369 (46.2)	772 (44.6)	540 (43.0)	0.34 ^b^	
Heart disease, no (%)	585 (15.5)	131 (16.4)	260 (15.0)	194 (15.4)	0.67 ^b^	
Diabetes, no (%)	499 (13.2)	101 (12.7)	233 (13.5)	165 (13.1)	0.86 ^b^	
Cognitive function						
MMSE, score *	27.0 (25.0–29.0)	27.0 (25.0–28.0)	27.0 (25.0–29.0)	27.0 (25.0–29.0)	0.06 ^a^	
Word memory, number *	11.3 (9.3–13.3)	11.3 (9.7–13.3)	11.3 (9.3–13.3)	11.3 (9.7–13.3)	0.73 ^a^	
TMT-A, sec. *	19.0 (17.0–23.0)	20.0 (17.0–23.0)	19.0 (17.0–23.0)	19.0 (16.0–23.0)	0.63 ^a^	
TMT-B, sec. *	38.0 (30.0–48.0)	38.0 (30.0–49.0)	38.0 (31.0–48.0)	37.0 (30.0–47.0)	0.22 ^a^	
SDST, number *	39.0 (34.0–44.0)	39.0 (34.0–44.0)	39.0 (34.0–44.0)	39.0 (34.0–45.0)	0.47 ^a^	
Lifestyle activity items, number *	9.0 (8.0–11.0)	9.0 (8.0–11.0)	9.0 (8.0–11.0)	9.0 (7.0–10.0)	0.01 ^a^	Very < Somewhat < Car
Using the bus and train, yes (%)	92.2	90.3 ^d^	91.8	93.9 ^c^	<0.05 ^b^	
Go to buy daily necessities, yes (%)	97.0	96.5	97.0	97.5	0.45 ^b^	
Engage in cash handling and banking, yes (%)	90.8	90.4	91.1	90.6	0.78 ^b^	
Go to a friend’s house, yes (%)	89.2	89.6	89.6	88.3	0.46 ^b^	
Drive a car, yes (%)	73.8	79.3 ^c^	74.4	69.6 ^d^	<0.001 ^b^	
Go out to throw the trash, yes (%)	86.1	85.2	85.9	86.9	0.51 ^b^	
Engage in fieldwork or gardening, yes (%)	74.3	79.0 ^c^	76.8 ^c^	68.0 ^d^	<0.001 ^b^	
Engage in hobbies or sports activities, yes (%)	77.6	77.4	77.4	78.0	0.91 ^b^	
Participate in events, yes (%)	51.9	55.4	51.0	50.9	0.08 ^b^	
Attend meetings in the community, yes (%)	53.9	60.7 ^c^	54.0	49.6 ^d^	<0.001 ^b^	
Use maps to go to unfamiliar places, yes (%)	65.4	63.1	67.1	64.7	0.11 ^b^	
Engage in cultural classes, yes (%)	44.4	41.3	45.9	44.4	0.10 ^b^	
Cognitive status						
NC, yes	2656 (70.2)	544 (68.2)	1219 (70.4)	893 (71.0)	0.36 ^b^	
MCI, yes	663 (17.5)	137 (17.2)	301 (17.4)	225 (17.9)	0.90 ^b^	
GCI, yes	467 (12.3)	117 (14.7)	211 (12.2)	139 (11.1)	0.05 ^b^	

* Median (IQR: Interquartile Range), MMSE: Mini-Mental State Examination; TMT: Trail Making Test; SDST: Symbol Digit Substitution Test; NC: normal condition; MCI: mild cognitive impairment; GCI: global cognitive impairment; Car: car-dependent; Somewhat: somewhat walkable; Very: very walkable. ^a^ *p*-Values reported from Kruskal–Wallis test, the Bonferroni correction for the Mann–Whitney U-test. ^b^ *p*-Values obtained by Pearson’s chi square test. ^c^ Statistically significant association by adjusted standardized residual >1.96 (*p* < 0.05). ^d^ Statistically significant association by adjusted standardized residual <−1.96 (*p* < 0.05).

**Table 2:** Multinomial logistic regression analysis with cognitive status as the dependent variable.

	Unadjusted Model						Adjusted Model					
	MCI			GCI			MCI			GCI		
	OR	95% CI	*p* Value	OR	95% CI	*p* Value	OR	95% CI	*p* Value	OR	95% CI	*p* Value
Car-dependent	1.00	0.79, 1.27	0.99	1.38	1.06, 1.81	<0.05	0.99	0.78, 1.26	0.96	1.28	0.97, 1.69	0.08
Somewhat walkable	0.98	0.81, 1.19	0.84	1.11	0.88, 1.40	0.37	0.99	0.82, 1.20	0.92	1.10	0.86, 1.39	0.46
Very walkable	1.00			1.00			1.00			1.00		

Adjusted model is adjusted for age, sex, years of education, number of medications, hypertension, heart disease, diabetes, and number of lifestyle activities; CI: confidence interval; MCI: mild cognitive impairment; GCI: global cognitive impairment; OR: odds ratio.

**Table 3:** Demographic characteristics of the six groups combining neighborhood amenities status and lifestyle activity items.

	Total	Car-Dependent		Somewhat Walkable		Very Walkable		*p*-Value	Post Hoc
		Fewer Lifestyle Activity Items	More Lifestyle Activity Items	Fewer Lifestyle Activity Items	More Lifestyle Activity Items	Fewer Lifestyle Activity Items	More Lifestyle Activity Items		
Demographic characteristics									
Number (%)	3786	293 (7.7)	505 (13.3)	659 (17.4)	1072 (28.3)	510 (13.5)	747 (19.7)		
Age, year *	70.0 (68.0–75.0)	70.0 (67.0–75.0)	70.0 (67.0–74.0)	70.0 (67.0–75.0)	71.0 (68.0–75.0)	70.0 (67.0–75.0)	70.0 (68.0–74.0)	0.19 ^a^	
Sex, Female (%)	1948 (51.5)	156 (53.2)	246 (48.7)	348 (52.8)	522 (48.7)	281 (55.1)	395 (52.9)	0.11 ^b^	
Education, years *	12.0 (9.0–12.0)	10.0 (9.0–12.0)	12.0 (9.0–12.0)	12.0 (9.0–12.0)	12.0 (9.0–13.0)	11.0 (9.0–12.0)	12.0 (9.0–13.0)	<0.001 ^a^	CF < CM, SF, SM, VM; CM, SF VF < SM, VM
Medication, number *	1.0 (0.0–3.0)	1.0 (0.0–3.0)	1.0 (0.0–3.0)	2.0 (0.0–3.0)	1.0 (0.0–3.0)	2.0 (0.0–3.0)	1.0 (0.0–3.0)	0.003 ^a^	SM < SF, VF
Chronic disease									
Hypertension, yes (%)	1681 (44.4)	133 (45.4)	236 (46.7)	300 (45.5)	472 (44.0)	232 (45.5)	308 (41.2)	0.43 ^b^	
Heart disease, yes (%)	585 (15.5)	49 (16.7)	82 (16.2)	92 (14.0)	168 (15.7)	79 (15.5)	115 (15.4)	0.88 ^b^	
Diabetes, yes (%)	499 (13.2)	39 (13.3)	62 (12.3)	85 (12.9)	148 (13.8)	70 (13.7)	95 (12.7)	0.96 ^b^	
Cognitive function									
MMSE, score *	27.0 (25.0–29.0)	26.0 (24.0–28.0)	27.0 (25.0–28.0)	27.0 (25.0–28.0)	27.0 (25.0–29.0)	27.0 (25.0–28.0)	27.0 (25.0–29.0)	<0.001 ^a^	CF <SM, VM; SF < VM
Word memory, number *	11.3 (9.3–13.3)	11.0 (9.3–13.0)	11.7 (9.7–13.3)	11.3 (9.0–13.3)	11.7 (9.3–13.3)	11.3 (9.3–13.3)	11.7 (9.7–13.3)	0.12 ^a^	
TMT–A, sec. *	19.0 (17.0–23.0)	20.0 (17.0–24.0)	20.0 (17.0–23.0)	20.0 (17.0–23.0)	19.0 (17.0–22.0)	20.0 (17.0–25.0)	19.0 (16.0–22.0)	<0.001 ^a^	VM < CF, SF, VF; SM < SF, VF
TMT–B, sec. *	38.0 (30.0–48.0)	40.0 (32.0–55.0)	36.0 (30.0–46.5)	40.0 (32.0–50.0)	36.5 (30.0–47.0)	38.0 (31.0–50.3)	36.0 (29.0–46.0)	<0.001 ^a^	CM, SM, VM < CF, SF; VM < SF
SDST, number *	39.0 (34.0–44.0)	38.0 (32.0–42.0)	39.0 (35.0–45.0)	38.0 (33.0–43.0)	40.0 (35.0–45.0)	38.0 (33.0–43.0)	40.0 (35.0–46.0)	<0.001 ^a^	CF, SF, VF < CM, SM, VM
Cognitive status									
NC, yes (%)	2656 (70.2)	177 (60.4) ^d^	367 (72.7)	434 (65.9) ^d^	785 (73.2) ^c^	348 (68.2)	545 (73.0)	<0.001 ^b^	
MCI, yes (%)	663 (17.5)	64 (21.8) ^c^	73 (14.5)	127 (19.3)	174 (16.2)	94 (18.4)	131 (17.5)	0.08 ^b^	
GCI, yes (%)	467 (12.3)	52 (17.7) ^c^	65 (12.9)	98 (14.9) ^c^	113 (10.5) ^d^	68 (13.3)	71 (9.5) ^d^	<0.01 ^b^	

* Median (IQR: interquartile range), MMSE: Mini-Mental State Examination; TMT: Trail Making Test; SDST: Symbol Digit Substitution Test; NC: normal condition; MCI: mild cognitive impairment; GCI: global cognitive impairment; CF: car dependent group with fewer lifestyle activities; CM: car dependent group with more lifestyle activities; SF: somewhat walkable group with fewer lifestyle activities; SM: somewhat walkable group with more lifestyle activities; VF: very walkable group with fewer lifestyle activities; VM: very walkable group with more lifestyle activities. ^a^ *p*-Values reported from Kruskal–Wallis test, the Bonferroni correction for the Mann–Whitney U-test. ^b^ *p*-Values obtained by Pearson’s chi square test. ^c^ Statistically significant association by adjusted standardized residual > 1.96 (*P* < 0.05). ^d^ Statistically significant association by adjusted standardized residual <−1.96 (*p* < 0.05).

## Supplementary Materials

The following are available online at https://www.mdpi.com/2077-0383/9/7/2109/s1, Table S1: Demographic Characteristics of Older Adults from Each Cognitive Status Group.
