# Supplementary material for: The Association between Neighborhood Amenities and Cognitive Function: Role of Lifestyle Activities
Source: J Clin Med. 2020 Jul 4;9(7):2109. doi: 10.3390/jcm9072109 (PMC7408849; doi:10.3390/jcm9072109)
Supplement: Supplementary file 1 [file jcm-09-02109-s001.pdf]

Supplementary Table 1. Demographic Characteristics of Older Adults from Each Cognitive Status Group

	NC n = 2656 (70.2)	MCI n =663 (17.5)	GCI n = 467 (12.3)	p value	Post-hoc
**Demographic characteristics**					
Age, year*	70.0 (67.0-74.0)	71.0 (68.0-75.0)	72.0 (69.0-77.0)	<0.001[a]	NC < MCI < GCI
Sex, Female (%)	1420 (53.5)[c]	349 (52.6)	179 (38.3)[d]	<0.001[b]	
Education, years*	12.0 (9.0-13.0)	12.0 (9.0-12.0)	9.0 (9.0-12.0)	<0.001[a]	GCI < MCI < NC
Medication, number*	1.0 (0.0-3.0)	2.0 (0.0-3.0)	2.0 (0.0-3.0)	0.002[a]	NC < MCI
**Chronic disease**					
Hypertension, no (%)	1505 (56.7)	349 (52.6)	251 (53.7)	0.12[b]	
Heart disease, no (%)	2248 (84.6)	555 (83.7)	398 (85.2)	0.77[b]	
Diabetes, no (%)	2311 (87.0)	567 (85.5)	409 (87.6)	0.52[b]	
**Cognitive function**					
MMSE, score*	28.0 (26.0-29.0)	26.0 (25.0-28.0)	23.0 (22.0-23.0)	<0.001[a]	GCI < MCI < NC
Word memory, number*	12.0 (10.3-13.7)	10.0 (7.7-12.0)	9.3 (7.0-11.7)	<0.001[a]	GCI < MCI < NC
TMT-A, sec.*	19.0 (16.0-21.0)	24.0 (19.0-29.0)	21.0 (18.0-26.0)	<0.001[a]	NC < GCI < MCI
TMT-B, sec.*	35.0 (29.0-42.0)	55.0 (40.0-73.0)	47.0 (37.0-65.0)	<0.001[a]	NC < GCI < MCI
SDST, number*	41.0 (36.0-46.0)	34.0 (29.0-39.0)	35.0 (29.0-40.0)	<0.001[a]	GCI, MCI < NC
Lifestyle activity items, number*	9.0 (8.0-11.0)	9.0 (7.0-11.0)	9.0 (7.0-10.0)	<0.001[a]	GCI, MCI < NC
Using the bus and train, yes (%)	2461 (92.7)	600 (90.6)	427 (91.4)	0.16[b]	
Go to buy daily necessities, yes (%)	2583 (97.3)	637 (96.2)	450 (96.6)	0.27[b]	
Engage in cash handling and banking, yes (%)	2452 (92.4)[c]	587 (88.9)	395 (84.6)[d]	<0.001[b]	
Go to a friend's house, yes (%)	2394 (90.2)[c]	577 (87.2)	403 (86.3)[d]	0.01[b]	
Drive a car, yes (%)	2007 (75.7)[c]	456 (68.9)[d]	326 (70.0)[d]	<0.001[b]	
Go out to throw the trash, yes (%)	2282 (86.0)	573 (86.7)	399 (85.6)	0.86[b]	
Engage in fieldwork or gardening, yes (%)	1977 (74.5)	494 (74.8)	338 (72.5)	0.63[b]	
Engage in hobbies or sports activities, yes (%)	2107 (79.5)[c]	494 (74.7)[d]	331 (70.9)[d]	<0.001[b]	
Participate in events, yes (%)	1391 (52.5)	335 (50.7)	234 (50.3)	0.55[b]	
Attend meetings in the community, yes (%)	1429 (54.0)	375 (56.6)	232 (49.8)	0.08[b]	
Use maps to go to unfamiliar places, yes (%)	1773 (67.0)[c]	406 (61.2)[d]	291 (62.3)	0.01[b]	
Engage in cultural classes, yes (%)	1227 (46.3)[c]	283 (42.7)	167 (35.9)[d]	<0.001[b]	

* Median (IQR: Interquartile Range), MMSE: Mini-Mental State Examination; TMT: Trail Making Test; SDST: Symbol Digit Substitution Test, NC: normal cognition; MCI: mild cognitive impairment; GCI: global cognitive impairment. [a] p values reported from Kruskal–Wallis test, the Bonferroni correction for the Mann–Whitney U-test. [b]p values obtained by Pearson's chi square test. [c]Statistically significant association by adjusted standardized residual > 1.96 ($p < 0.05$). [d]Statistically significant association by adjusted standardized residual < −1.96 ($p < 0.05$).
